# The effect of orthognathic surgery on the lip lines while smiling in skeletal class III patients with facial asymmetry

**DOI:** 10.1186/s40902-016-0065-1

**Published:** 2016-03-31

**Authors:** Sang-Hoon Kang, Moon-Key Kim, Sang-In An, Ji-Yeon Lee

**Affiliations:** 1 Department of Oral and Maxillofacial Surgery, National Health Insurance Service Ilsan Hospital, 100 Ilsan-ro, Ilsan-donggu, Goyang, Gyeonggi-do 410-719 Republic of Korea; 2 Department of Orthodontics, National Health Insurance Service Ilsan Hospital, 100 Ilsan-ro, Ilsan-donggu, Goyang, Gyeonggi-do 410-719 Republic of Korea

**Keywords:** Facial asymmetry, Orthognathic surgery, Lip cant, Smile

## Abstract

**Background:**

The aim of this study was to examine the relationship between improvements in lip asymmetry at rest and while smiling after orthognathic surgery in patients with skeletal class III malocclusion.

**Methods:**

This study included 21 patients with skeletal class III malocclusion and facial asymmetry. We used preoperative and postoperative CT data and photographs to measure the vertical distance of the lips when smiling. The photographs were calibrated based on these distances and the CT image. We compared preoperative and postoperative results with the *t* test and correlations between measurements at rest and when smiling by regression analyses.

**Results:**

There were significant correlations between the postoperative changes in canting of the mouth corners at rest, canting of the canines, canting of the first molars, the slope of the line connecting the canines, and the slope of the line connecting first molars. The magnitude of the postoperative lip line improvement while smiling was not significantly correlated with changes in the canting and slopes of the canines, molars, and lip lines at rest.

**Conclusions:**

It remains difficult to predict lip line changes while smiling compared with at rest after orthognathic surgery in patients with mandibular prognathism, accompanied by facial asymmetry.

## Background

Patients with craniofacial asymmetry frequently also have lip line asymmetry [1]. Asymmetry leads to canted lips due to a difference in oral commissure. It is of interest to both patients and surgeons whether lip asymmetry can be corrected through orthognathic surgery. It has been reported that the improvement in lip canting can be proportional to the jaw displacement correction [1–3]. However, lip lines, which are important for the patient’s esthetic satisfaction, are affected by several factors such as the properties of soft tissues and the patient’s muscular activity [4]. Thus, it is difficult to accurately predict the changes that will occur postoperatively. Unfortunately, there has been little research in this area, especially in lip asymmetry while smiling [4, 5]. Lip asymmetry when smiling does not usually occur in connection with craniofacial asymmetry [5].

Therefore, the present study examined the relationship between improvements in lip asymmetry at rest and when smiling after orthognathic surgery in patients with skeletal class III malocclusion and menton deviation.

## Methods

Our institutional review board approved this retrospective study. The subjects were patients with mandibular prognathism accompanied by facial asymmetry. More specifically, the subjects comprised patients with skeletal class III malocclusion, a menton displaced laterally more than 2 mm, and no maxillary retrusion. All of the patients underwent bimaxillary orthognathic surgery, namely a Le Fort I maxillary osteotomy and mandibular vertical ramus osteotomy. Patients were only included if they finished preoperative orthodontic treatment, underwent orthognathic surgery, had preoperative and postoperative CT data, and had clinical photographs of themselves smiling that were taken after the surgery, and postoperative orthodontic treatments had been concluded. Twenty-one patients met the above criteria and were included.

### Reference planes for measurements on CT images

**Fig. 1:**
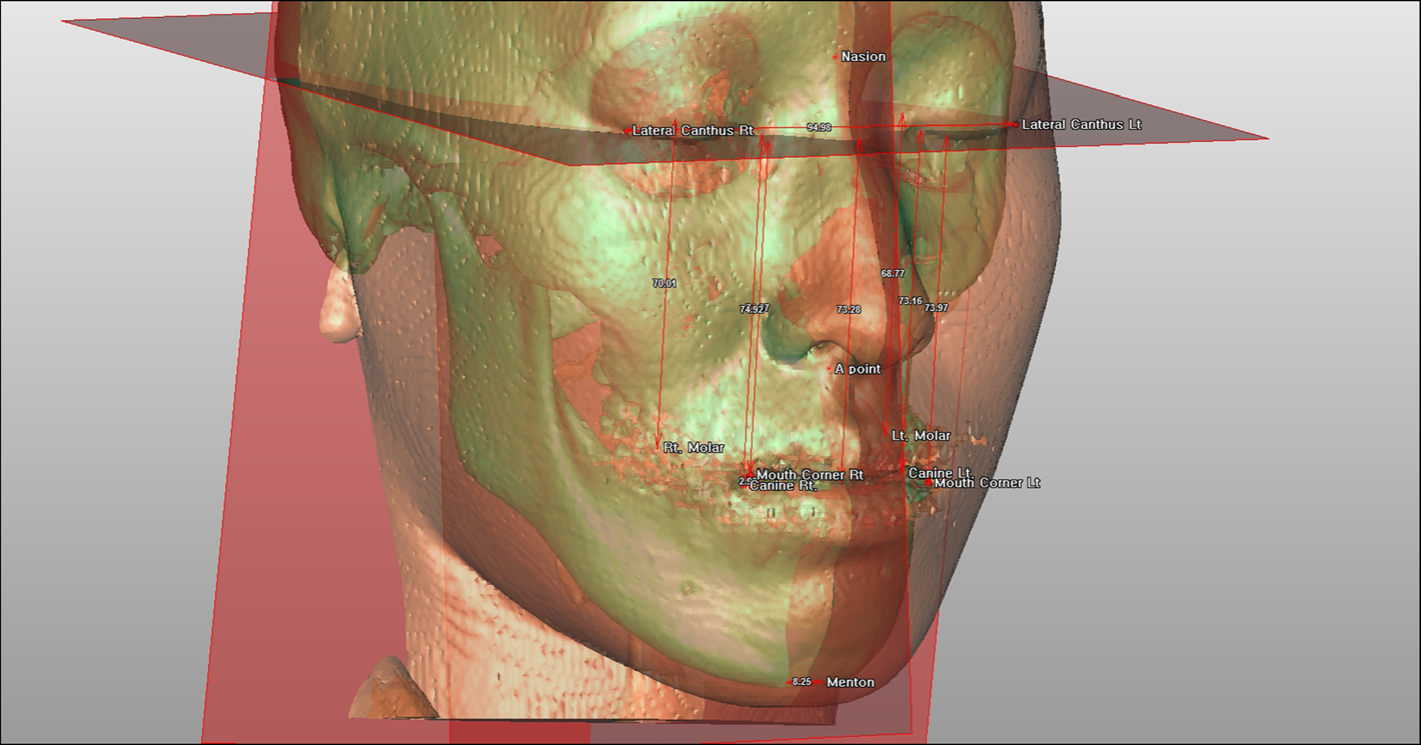
Reference planes, points, and measurements in three-dimensional CT images

We collected the preoperative and postoperative CT data, and 3D images were reconstructed using SIMPLANT® software ver. 14.0 (Materialise Dental, Leuven, Belgium). Then, we used the constructed 3D images and three surface images to measure error. The errors were measured as follows. First, we set reference planes in the cranial area of the data. Then, we measured each point on the reconstructed mandible and on the surgical simulation and compared their distances. We constructed three reference planes: the Frankfort horizontal (FH), midsagittal, and coronal. The FH plane was used to measure vertical error. The FH plane crossed the center of the orbitale―the infraorbital margins―and the two portions of both external auditory canals. The plane that runs parallel to the FH plane and crossed the center of the lateral canthus was set as the intercanthus horizontal plane. The midsagittal plane was used to measure lateral error and was perpendicular to the FH plane and crossed the nasion and internal occipital crest. Finally, we used the coronal plane to measure anteroposterior error. It ran perpendicular to the FH plane and midsagittal plane and crossed the nasion. The shortest distances from each of these three planes were named the vertical distance, lateral distance, and anteroposterior distance (Fig. 1).

### Setting reference points and measurements in CT images

We measured the vertical distances from the left and right canines, molars, and mouth corners to the intercanthus horizontal plane. The differences in the heights of the left and right canines, molars, and mouth corners were defined as canting. The angles between the lines that connect the left and right canines, molars, mouth corners and the horizontal plane were defined as canine, molar, and mouth corner angles. The distances from the coronal plane to point A and point B were measured as the horizontal distance A and horizontal distance B, respectively. The distance from the midsagittal plane to the menton was set as the menton deviation. We measured each of these distances on the preoperative and postoperative CT data. The measurement points and measurement methods are shown in Table 1.

**Table 1:** Descriptions of measurements

Name	Descriptions
Point A (mm)	Distance to point A from the coronal plane
Point B (mm)	Distance to point B from the coronal plane
Menton deviation (mm)	Distance to the menton point from the midsagittal plane
Lateral intercanthus (mm)	Distance between the right lateral canthus point and left lateral canthus point
Right canine (mm)	Distance to the right canine cusp from the intercanthus horizontal plane
Left canine (mm)	Distance to the left canine cusp from the intercanthus horizontal plane
Right molar (mm)	Distance to the right first molar alveolar bone from the intercanthus horizontal plane
Left molar (mm)	Distance to the left first molar alveolar bone from the intercanthus horizontal plane
Right mouth corner (mm)	Distance to the right mouth commissure from the intercanthus horizontal plane
Left mouth corner (mm)	Distance to the left mouth commissure from the intercanthus horizontal plane
Molar canting (mm)	Absolute value of the height difference between the right first molar point and left first molar point
Canine canting (mm)	Absolute value of the height difference between the right canine point and left canine point
Mouth corner canting (mm)	Absolute value of the height difference between the right mouth corner and left mouth corner
Canine line (°)	Absolute value of the angle between the line connecting both canines and the intercanthus horizontal plane
Molar line (°)	Absolute value of the angle between the line connecting both first molars and the intercanthus horizontal plane
Mouth corner line (°)	Absolute value of the angle between the line connecting both mouth corners and the intercanthus horizontal plane
Smiling right mouth corner (mm)	Distance to the right mouth commissure from the intercanthus horizontal plane, when smiling
Smiling left mouth corner (mm)	Distance to the left mouth commissure from the intercanthus horizontal plane, when smiling
Smiling mouth corner canting (mm)	Absolute value of the height difference between the right mouth corner and left mouth corner, when smiling
Smiling mouth corner line (°)	Absolute value of the angle between the line connecting both mouth corners and the intercanthus horizontal plane, when smiling

### Measurements of lip lines while smiling

**Fig. 2:**
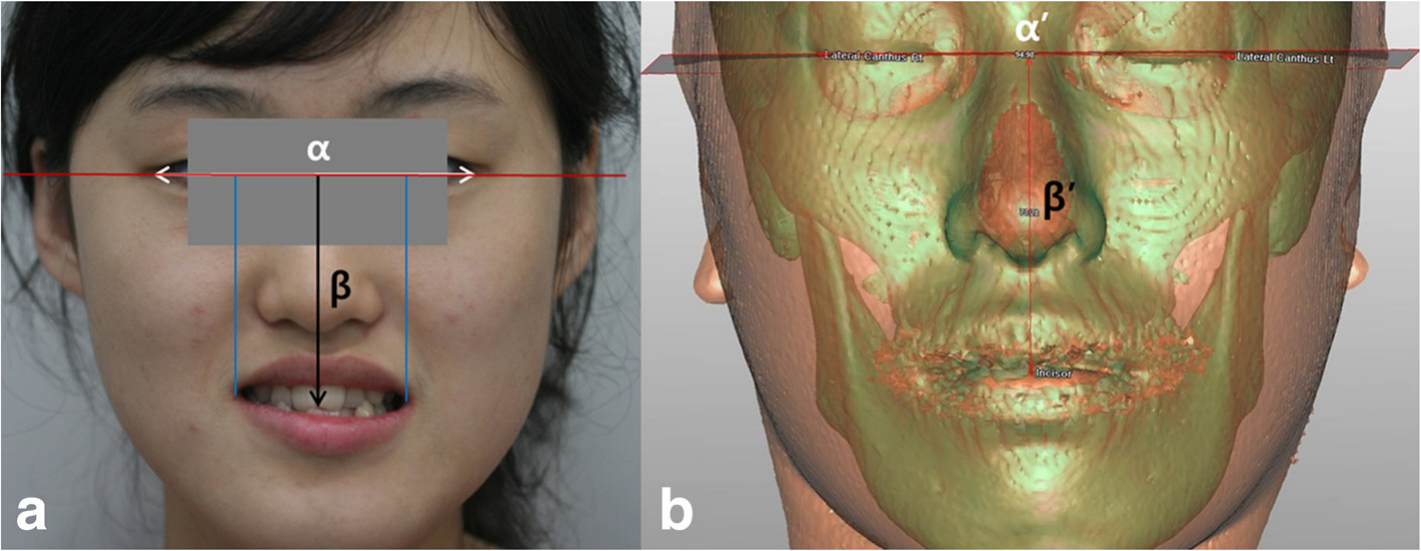
Calibration of a clinical picture of a patient’s smile according to the 3D CT image (e.g., preoperative image). **a** 2D photograph; **b** 3D CT image. At first, the lateral intercanthus distance (*white α*) in the smile picture matched that (*white α’*) in the 3D CT image. The vertical ratio of the photograph was modified by adjusting the vertical distance (*black β*) from the lateral intercanthus line to the anterior tooth in the smile picture to match that (*black β’*) in the 3D CT image. The vertical distance of the lips during smiling, which is the shortest distance (*blue line*) from the lateral intercanthus line (*red line*) to the mouth corner in the photograph

We used the preoperative and postoperative photographs of the patients while smiling to measure the difference in the vertical distance of the lips. At first, the observer adjusted the whole size of photographs according to the distant reference of CT. The photographs were collected using V-Ceph ver. 6.0 (OSSTEM IMPLANT Co., Seoul, Republic of Korea) software. The same program was used to measure the patients’ right and left lateral canthus distances (lateral intercanthus distance). The preoperative photograph was adjusted based on these distances and the lateral intercanthus distance that was measured on the preoperative CT image (Fig. 2a). At second, using the firstly revised picture, we measured the vertical distance from the line (lateral intercanthus line) that connects the right and left lateral canthus to the edge of the maxillary central incisors in V-Ceph. We also measured the vertical distance from the edge of the maxillary central incisors to the intercanthus horizontal plane on the preoperative CT. Then finally, we altered the vertical size of the smile picture (the firstly revised picture) that was adjusted using the intercanthus distance to have the same vertical distance from the lateral intercanthus line to the central incisors as those vertical distance from the intercanthus horizontal plane to the central incisors measured on the preoperative CT data (Fig. 2b). The alteration of the photograph was completed in the state of the same value of the lateral intercanthus distance and the vertical distance from incisor to the lateral intercanthus line.

We measured the vertical distance of the lips during smiling, which is the shortest distance from the lateral intercanthus line to the mouth corner. The difference between the right and left vertical distances was defined as canting. The angle between the line that connects the left and right mouth corners and the lateral intercanthus line was defined as the slope angle while smiling. We acquired preoperative and postoperative data by editing the preoperative and postoperative smile pictures via the same procedure. We also calculated the magnitude of change after surgery.

### Measurement of error induced by the photo editing process

We randomly selected ten patients from the sample of 21 subjects. Their preoperative pictures of mouths at rest were modified using the same calibrating procedure used for the smiling pictures. The revised pictures were used to measure the vertical distances of the lips at rest. We used the V-Ceph ver. 6.0 (OSSTEM IMPLANT Co.) software to measure the right and left lateral canthus distances of the patients’ lips at rest. Then, we adjusted the lateral intercanthus distance of the photograph to that measured on the preoperative CT data.

Using the revised picture, we measured the vertical distance from the line that connects the left and right lateral canthus to the right mouth corner. We also measured the vertical distance from the lateral canthus plane to the right mouth corner on the preoperative CT data. Then, we modified the vertical ratio of the resting picture that was adjusted using the lateral intercanthus distance to match the distance from the intercanthus line to the right mouth corner of the picture and the preoperative CT. After these modifications, the lateral intercanthus distance and distance from the lateral intercanthus line to the central incisors were the same as those measured on the preoperative CT data. Then, using the revised picture, we measured the vertical distance from the lateral intercanthus line to the right mouth corner. We also measured the vertical distance from the intercanthus horizontal plane to the right mouth corner on the CT image and defined the absolute values of this difference in distance as the error of the clinical photographs. One time per patient, one observer measured the distance in the image of the patients.

### Intraoperator error

We used the ten randomly selected patients for measuring errors for clinical photo adjustment. To measure the intraoperator error, one observer measured the vertical distance from the intercanthus line to the right mouth corner in these pictures and measured the errors in the reference points during each trial one time per patient.

The intraoperative error during CT image measurements was measured using preoperative CT images of the same ten patients. The intraoperative error during CT image measurements was defined as the difference in the distances from the intercanthus horizontal plane to the right mouth corner when repeatedly measured in ten patients.

### Statistical analysis

We compared preoperative and postoperative results by Wilcoxon signed-rank test. The correlations among preoperative and postoperative canting of the canines, canting of the molars, slope of the canines, slope of the molars, canting of the lips at rest, and slope of the lips at rest were examined by regression analyses. Similarly, the correlations among preoperative and postoperative canting of the canines, canting of the molars, slope of the canines, slope of the molars, and canting and slope angle of the lips while smiling were analyzed by regression analyses. The statistical significances of the results were verified using the IBM SPSS statistics 21 (IBM Corp., Armonk, NY, USA) program. The level of significance was set at 0.05.

## Results

There were 21 subjects, 11 females and 10 males in this study. The mean age (mean ± standard deviation (SD)) was 22.0 ± 5.0 years. The average period of time from the surgery to the completion of the postoperative orthodontic treatments, photographs, and CT scans (mean ± SD) was 19.0 ± 10.2 months. After the surgery, the mandibular position of the patients was displaced to the posterior and the horizontal distance from the coronal plane to point B was reduced from a mean of 91.22 mm before surgery to a mean of 82.41 mm after surgery (*P* = <.001). The lateral displacement of the menton was also reduced from a mean of 4.54 mm before surgery to a mean of 1.50 mm after surgery (*P* = .001).

The mean vertical distances at the left and right first molars before surgery were 56.82 and 56.71 mm, respectively, and they were reduced to 54.06 and 54.54 mm, respectively, after surgery. The mean canting of the canines was 1.06 mm (range, 0.12 to 3.85 mm) before surgery and (range, 0.01 to 2.99 mm) after surgery. The mean canting of the molar areas was 1.40 mm (range, 0.30 to 3.67 mm) before surgery and 1.36 mm (range, 0.18 to 3.86 mm) after surgery, but the difference was not significant. The canting of the mouth corners at rest was reduced from 1.54 mm (range, 0.19 to 5.58 mm) before surgery to 0.94 mm (range, 0.08 to 2.54 mm) after surgery (*P* = .048). The canting of the mouth corners while smiling was increased from 1.09 mm (range, 0.09 to 4.45 mm) before surgery to 1.30 mm (range, 0.04 to 3.78 mm) after surgery.

The angle of the line that connects the first molars was slightly reduced from 1.44° before surgery to 1.40° after surgery, but the difference was not significant. Similarly, the angle of the line that connects the canines was slightly reduced from 1.75° before surgery to 1.71° after surgery, but the difference was not significant. The angle of the line connecting the mouth corners at rest was also reduced from 0.70° before surgery to 0.56° after surgery. Finally, the angle of the line connecting the mouth corners while smiling was increased from 1.19° before surgery to 1.42° after surgery (Table 2). The mean difference in canting of the canines and first molars was about 1.4 mm. The mean difference in canting of the lips while smiling was about 1.2 mm. The mean differences in the angles of the lines that connect the canines and first molars were 2.3° and 1.4°, respectively. The mean differences in the angles of the lips at rest and while smiling were 0.6° and 1.4°, respectively (Table 2).

**Table 2:** Measurement results pre-, post-surgery, and differences (mean ± SD)

Measurement	Pre-surgery	Post-surgery	*P* value^a^	Differences
Point A (mm)	87.37 ± 4.69	88.10 ± 4.41	.821	−0.73 ± 1.95
Point B (mm)	91.22 ± 8.98	82.41 ± 6.55	<.001*	8.81 ± 4.26
Menton deviation (mm)	4.54 ± 3.92	1.50 ± 1.22	.001*	3.04 ± 3.76
Right canine (mm)	68.96 ± 3.90	69.08 ± 3.57	.768	−0.11 ± 1.79
Left canine (mm)	69.18 ± 3.63	68.74 ± 3.57	.394	0.44 ± 2.18
Right molar (mm)	56.71 ± 3.88	54.54 ± 3.46	.001*	2.16 ± 2.38
Left molar (mm)	56.82 ± 3.67	54.06 ± 3.62	.001*	2.76 ± 2.53
Right mouth corner (mm)	67.74 ± 4.23	69.51 ± 4.14	<.001*	−1.77 ± 1.74
Left mouth corner (mm)	67.72 ± 4.36	69.22 ± 4.01	.005*	−1.50 ± 2.00
Canine canting (mm)	1.06 ± 0.85	1.06 ± 0.75	.664	1.44 ± 1.08^b^
Molar canting (mm)	1.40 ± 1.06	1.36 ± 0.97	.945	1.42 ± 1.16^b^
Mouth corner canting (mm)	1.54 ± 1.26	0.94 ± 0.58	.048*	1.28 ± 1.03^b^
Canine line (°)	1.75 ± 1.44	1.71 ± 1.22	.794	2.32 ± 1.70^b^
Molar line (°)	1.44 ± 1.12	1.40 ± 1.01	.931	1.44 ± 1.17^b^
Mouth corner line (°)	0.70 ± 0.49	0.56 ± 0.42	.370	0.66 ± 0.65^b^
Smiling right mouth corner (mm)	66.39 ± 4.58	64.59 ± 4.47	.020*	1.8 ± 3.21
Smiling left mouth corner (mm)	66.09 ± 4.79	64.36 ± 4.58	.092	1.73 ± 3.52
Smiling mouth corner canting (mm)	1.09 ± 1.03	1.30 ± 1.06	.322	1.27 ± 1.13^b^
Smiling mouth corner line (°)	1.19 ± 1.06	1.42 ± 1.20	.588	1.43 ± 1.26^b^

*Differences* change between the preoperative state and postoperative result (preoperative value-postoperative value), *SD* standard deviation

*P < .05

^a^Significance according to the Wilcoxon signed-rank test

^b^Absolute value of difference

There was a significant correlation between the magnitudes of the postoperative changes in the canting of the mouth corners at rest and in the canting of the canines (*P* = .009). Similarly, there was also a significant correlation between the magnitudes of the postoperative changes in the canting of the mouth corners at rest and in the canting of the first molars (*P* = .023). In addition, there was a significant correlation between the extent of the postoperative change in the canting of the mouth corners at rest and the change in the slope of the line that connects both canines (*P* = .010). Similarly, there was a significant correlation between the magnitude of the postoperative change in the canting of the mouth corners at rest and the change in the slope of the line that connects both first molars (*P* = .017) (Table 3).

**Table 3:** Regression analysis of mouth corner canting (mm) according to the changes in the canine and molar areas

	*A*	*P* value^a^	*B*	*P* value^b^	*β*	*R* ^2^	*P* value^c^
Canine canting (mm)	0.535	.009*	0.509	.136	0.558	0.311	.009*
Molar canting (mm)	0.438	.023*	0.658	.056	0.494	0.244	.023*
Canine line (°)	0.334	.010*	0.507	.143	0.551	0.304	.010*
Molar line (°)	0.453	.017*	0.628	.064	0.515	0.265	.017*

*A* non-standardized regression coefficient, *B* constant, *β* standardized regression coefficient, *R* ^*2*^ coefficient of determination

*P < .05

^a^Significance of non-standardized regression coefficient

^b^Significance of constant

^c^Significance of regression analysis

However, there was no significant correlation between the changes in the slope of the line that connects the mouth corners at rest and the lines that connect the canines and connect the molars. Similarly, there was no significant correlation between the change in the slope of the line that connects the mouth corners at rest and the postoperative changes in canting of the canines and the molars (Table 4). The magnitude of the postoperative change in canting of the mouth corners when smiling was not significantly correlated with the extent of the postoperative changes in the canting and the slopes of the canines, molars, and lips at rest (Table 5). Similarly, the magnitude of the postoperative change in the slope angles of the mouth corners while smiling was not significantly correlated with the change in the canting and the slopes of the canines, molars, and lips at rest (Table 6).

**Table 4:** Regression analysis of the mouth corner line (°) according to the changes in the canine and molar areas

	*A*	*P* value^a^	*B*	*P* value^b^	*β*	*R* ^2^	*P* value^c^
Canine canting (mm)	−0.128	.358	0.850	.002*	−0.211	0.045	.358
Molar canting (mm)	−0.125	.330	0.844	.002*	−0.224	0.050	.330
Canine line (°)	−0.081	.359	0.853	.002*	−0.211	0.045	.359
Molar line (°)	−0.133	.295	0.858	.001*	−0.240	0.058	.295

*A* non-standardized regression coefficient, *B* constant, *β* standardized regression coefficient, *R* ^*2*^ coefficient of determination

*P < .05

^a^Significance of non-standardized regression coefficient

^b^Significance of constant

^c^Significance of regression analysis

**Table 5:** Regression analysis of mouth corner canting (mm) when smiling according to the changes in the canine and molar areas

	*A*	*P* value^a^	*B*	*P* value^b^	*β*	*R* ^2^	*P* value^c^
Canine canting (mm)	0.178	.464	1.019	.027*	0.169	0.029	.464
Molar canting (mm)	0.229	.305	0.950	.027*	0.235	0.055	.305
Mouth corner canting (mm)	0.023	.929	1.248	.007*	0.021	<0.001	.929
Canine line (°)	0.115	.455	1.011	.030*	0.172	0.030	.455
Molar line (°)	0.216	.330	0.965	.026*	0.224	0.050	.330
Mouth corner line (°)	0.103	.799	1.209	.004*	0.059	0.004	.799

*A* non-standardized regression coefficient, *B* constant, *β* standardized regression coefficient, *R* ^*2*^ coefficient of determination

*P < .05

^a^Significance of non-standardized regression coefficient

^b^Significance of constant

^c^Significance of regression analysis

**Table 6:** Regression analysis of the mouth corner line (°) when smiling according to the changes in the canine and molar areas

	*A*	*P* value^a^	*B*	*P* value^b^	*β*	*R* ^2^	*P* value^c^
Canine canting (mm)	0.091	.736	1.303	.014*	0.078	0.006	.736
Molar canting (mm)	0.277	.263	1.040	.439	.256	0.065	.263
Mouth corner canting (mm)	−0.099	.727	1.563	.003*	−0.081	0.007	.727
Canine line (°)	0.064	.709	1.287	.015*	0.087	0.007	.709
Molar line (°)	0.262	.286	1.057	.027*	0.244	0.060	.286
Mouth corner line (°)	0.075	.867	1.386	.003*	0.039	0.002	.867

*A* non-standardized regression coefficient, *B* constant, *β* standardized regression coefficient, *R* ^*2*^ coefficient of determination

*P < .05

^a^Significance of non-standardized regression coefficient

^b^Significance of constant

^c^Significance of regression analysis

The intraoperator error for the clinical pictures, which was measured by comparing the vertical distance measurements of the mouth corners, was 0.17 ± 0.11 mm (mean ± SD). The intraoperator error for the CT data, which was measured by comparing the vertical distance measurements of the mouth corners, was 0.27 ± 0.16 mm (mean ± SD). The error induced by photo adjustment, which was measured by comparing the values of the canting of the lips at rest on the CT and clinical pictures, was 0.63 ± 0.53 mm (mean ± SD).

## Discussion

As mentioned in the “Results” section, the postoperative differences in the canting and slope angles of the mouth corners while smiling were not significantly correlated with the postoperative differences in the canting and the slopes of the canines, molars, and lip line at rest. However, there was a significant correlation between the postoperative difference in canting of the mouth corners at rest and the change in the slope of the line that connects the canines.

A previous study that used soft tissue scanners and CT data reported there was a significant relationship between the degree of vertical positional change of the jaws and that of the lips after orthognathic surgery in patients with mandibular prognathism and facial asymmetry [2]. Similar results were also found in the present study. After orthognathic surgery in the canines and first molars, the degree of vertical positional changes of the jaw and lips were significantly correlated. However, after the same surgery, the degree of vertical positional change of the jaw was not significantly correlated with that of the lips while smiling.

The results of the present study show that orthognathic surgery results in different degrees of improvement of lip asymmetry while smiling and at rest. Benson and Laskin reported that lip canting while smiling may be different from the occlusal level in subjects without clinical facial asymmetry [5]. In addition, another study reported that the magnitude of facial expressions, including maximum smiling, were not significantly changed after orthognathic surgery for patients with facial asymmetry [4]. The position of the muscles may be changed through orthognathic surgery, but it is difficult to accurately predict the direction of this change [4, 5]. Lip asymmetry while smiling may result from activities of the zygomaticus major muscle [6]. Furthermore, muscle activity while smiling may be affected by psychological factors [7]. Therefore, an evaluation of the sizes and positions of facial muscles and an electromyography may be required for more accurate prediction of the results from orthognathic surgeries [6]. Thus, in agreement with the current study, previous reports suggested that lip asymmetry when smiling may not coincide with lip asymmetry at rest and may not be improved by orthognathic surgery.

The present study employed clinical photographs to assess lip line canting while smiling, which is one of the limitations. Smiling is not a static phenomenon. Predictability may be unclear in assessing the smile, as the precise anatomic location of many of their points could be significantly altered on the basis of muscle tone related to smiling and factors such as response to light (photographic flash), time of day, etc. We calibrated the length of the clinical photos based on our CT data by adjusting the distance ratio of the photo in accordance with the CT data. This process may have produced errors. The absolute value of the mean error that occurred during the calibrating process was 0.63 mm. There also may have been an error while measuring the lips during smiling from clinical photographs. Also, calibration method adjusted for the degree of relative magnification but would not necessarily adjust for dynamic changes related to muscle tone.

We conducted analysis on a mixture of images; one was 3D data and the others were 2D photographs. Measuring lip smile from 2D photographs is inaccurate and does not represent the 3D nature of this facial expression. Standardization is difficult in defining for facial expression before and after surgery; this may be essential to provide the corrected measurements. Canting of the lip corners will depend on the magnitude and extent of the smile; this has to be standardized, and evidence should be provided regarding the reproducibility of this facial expression. The authors agree that this may be a crucial factor in this study; without standardization of facial expressions, it would be almost impossible to measure dynamic soft tissue changes and relate them to the performed surgery.

To overcome these limitations of using clinical photographs for measurements, researchers may use facial scanners to acquire 3D images of the facial soft tissues or use two cameras to form a 3D image of the face [2, 4]. That being said, 3D images of faces have been used to verify the changes in soft tissues and the facial skeleton related to orthognathic surgeries [8, 9]. Additionally, postoperative swelling, particularly in the midface and perioral regions, could substantially affect the magnitude of soft tissue movements with smiling, as well as the surface features on a 2D photograph. In the current study, the average period of time from the surgery to the completion of the postoperative orthodontic treatments, photographs, and CT scans (mean ± SD) was 19.0 ± 10.2 months.

This study only involved the cases of patients with mandibular prognathism accompanied by facial asymmetry. For patients with maxillary or mandibular hypoplasia, orthognathic surgeries may significantly improve the magnitude of their facial expressions [2, 4, 10, 11]. We showed a negative result of study in a sample of 21 patients. We should have demonstrated quantitatively that our results are truly related to non-significance of the postulated associations, rather than a sample size issue. Further studies will examine the displacement of the jaw in orthognathic surgeries, including the pitch, roll, and yaw, as well as the changes of lip lines when smiling when the direction of lip canting changes after various surgeries [12]. We expect these studies to provide valuable knowledge for predicting the changes, induced by orthognathic surgery, in lip lines while smiling.

The mean value of lip canting was less than 2 mm, which was another limitation of this study. The mean lip canting value was also reduced from 1.54 mm before surgery to 0.94 mm after surgery. In addition, there were no large differences in the lip slopes before and after surgery (0.70° before surgery and 0.56° after surgery) and the slopes were also less than 1°, which was another limitation of this study. The threshold for the recognition of an occlusal cant was suggested to be 4° in a 2D frontal plane study [13, 14]. Most surgeons would agree that an occlusal cant of <3°–4° is likely not significant enough to pose a noticeable problem for a lay observer [13]. As such, it seems that the degree of lip asymmetry in the frontal plane among this cohort was relatively small at the level of occlusal plane. The manifest change at the level of the chin was more substantial but may differentially affect the lower lip soft tissues relative to the upper lip, influencing smiling in a less predictable way [15]. The present study did not clinically recognize occlusal cant or lip asymmetry; rather, it examined the correlation between lip asymmetry at rest and when smiling after orthognathic surgery. Further studies will be required to examine patients with more severe lip canting using various 3D imaging methods for clinical recognition of lip asymmetry [16–18].

The mean intraoperator error for the CT images was 0.27 mm, which was larger than that for the clinical pictures (0.17 mm). This is presumed to be due to a change of the horizontal plane, which was induced by an accumulation of intraoperator errors while setting anatomical structures required for the reference horizontal planes in the 3D CT images. The results of this study indicated that the magnitudes of the postoperative changes in the canting and the slope of the mouth corners at rest were significantly correlated with the changes in the canting and slopes of the canines and the molars. However, the magnitude of the postoperative improvement of the lip lines when smiling was not significantly correlated with the changes in the canting and slopes of the canines, molars, and lip lines at rest. Hence, it is still difficult to predict the changes in lip lines while smiling after an orthognathic surgery in patients with mandibular prognathism accompanied by facial asymmetry.

## Conclusions

In conclusion, the magnitude of the postoperative lip line improvement while smiling was not significantly correlated with changes in the canting and slopes of the canines, molars, and lip lines at rest. It remains difficult to predict lip line changes while smiling after orthognathic surgery in patients with mandibular prognathism, accompanied by facial asymmetry.

### Ethics statement/confirmation of patient’s permission

Ethical approval was provided by the Institutional Review Board of the NHIS Ilsan Hospital.
